# Treatment-Resistant Depression in a Real-World Setting: First Interim Analysis of Characteristics, Healthcare Resource Use, and Utility Values of the FondaMental Cohort

**DOI:** 10.3390/brainsci10120962

**Published:** 2020-12-10

**Authors:** Antoine Yrondi, Djamila Bennabi, Emmanuel Haffen, Delphine Quelard, Ludovic Samalin, Julia Maruani, Etienne Allauze, Damien Pierre, Thierry Bougerol, Vincent Camus, Thierry D’Amato, Olivier Doumy, Jérôme Holtzmann, Christophe Lançon, Fanny Moliere, Rémi Moirand, Isabel Nieto, Raphaëlle Marie Richieri, Mathilde Horn, Laurent Schmitt, Florian Stephan, Jean-Baptiste Genty, Guillaume Vaiva, Michel Walter, Philippe Courtet, Marion Leboyer, Pierre-Michel Llorca, Sophie Marguet, Nathalie Dennis, Dominique Schaetz, Wissam El-Hage, Bruno Aouizerate

**Affiliations:** 1 Fondation FondaMental, 94000 Créteil, France; djamila.bennabi@univ-fcomte.fr (D.B.); emmanuel.haffen@univ-fcomte.fr (E.H.); lsamalin@chu-clermontferrand.fr (L.S.); maruani.julia@gmail.com (J.M.); eallauze@chu-clermontferrand.fr (E.A.); damien_2903@hotmail.com (D.P.); TBougerol@chu-grenoble.fr (T.B.); vincent.camus@univ-tours.fr (V.C.); thierry.damato@ch-le-vinatier.fr (T.D.); odoumy@ch-perrens.fr (O.D.); jholtzmann@chu-grenoble.fr (J.H.); christophe.lancon@ap-hm.fr (C.L.); moliere.fanny@gmail.com (F.M.); remi.moirand@ch-le-vinatier.fr (R.M.); isabel.nieto@lrb.aphp.fr (I.N.); raphaellemarie.richieri@ap-hm.fr (R.M.R.); mathilde.horn@chru-lille.fr (M.H.); schmitt.l@chu-toulouse.fr (L.S.); florian.stephan@chu-brest.fr (F.S.); jeanbaptiste.genty@aphp.fr (J.-B.G.); guillaume.vaiva@chru-lille.fr (G.V.); michel.walter@chu-brest.fr (M.W.); philippe.courtet@univ-montp1.fr (P.C.); marion.leboyer@inserm.fr (M.L.); pmllorca@chu-clermontferrand.fr (P.-M.L.); wissam.elhage@univ-tours.fr (W.E.-H.); bruno.aouizerate@u-bordeaux.fr (B.A.); 2 ToNIC Toulouse NeuroImaging Center, Université de Toulouse, INSERM, UPS, 31059 Toulouse, France; 3 Service de Psychiatrie et de Psychologie Médicale, Centre Expert Dépression Résistante FondaMental, 31059 Toulouse, France; 4 Service de Psychiatrie Clinique, Centre Expert Dépression Résistante FondaMental, EA 481 Neurosciences, Université de Bourgogne Franche Comté, 25000 Besançon, France; 5 Service de Psychiatrie Clinique, Centre Expert Dépression Résistante FondaMental, Centre Investigation Clinique 1431-INSERM, EA 481 Neurosciences, Université de Bourgogne Franche Comté, 25000 Besançon, France; 6 Janssen, 97132 Issy-Les-Moulineaux, France; DQUELARD@ITS.JNJ.com (D.Q.); DSCHAETZ@its.jnj.com (D.S.); 7 Service de Psychiatrie de L’adulte B, Centre Expert Dépression Résistante FondaMental, CHU de Clermont-Ferrand, 63000 Clermont-Ferrand, France; 8 Service de Psychiatrie Adulte, Centre Expert Dépression Résistante FondaMental, Hôpital Fernand-Widal, 75010 Paris, France; 9 Service de Psychiatrie de L’adulte, Centre Expert Dépression Résistante FondaMental, Univ. Grenoble Alpes, Inserm, U1216, CHU Grenoble Alpes, Grenoble Institut Neurosciences, 38000 Grenoble, France; 10 Clinique Psychiatrique Universitaire, Centre Expert Dépression Résistante FondaMental, Inserm U1253 Imaging and Brain, CHRU de Tours, 37000 Tours, France; 11 Service Universitaire de Psychiatrie Adulte, Centre Expert Dépression Résistante FondaMental, Centre Hospitalier Le Vinatier, 69500 Bron CEDEX, France; 12 Pôle de Psychiatrie Générale et Universitaire, Centre Expert Dépression Résistante FondaMental, CH Charles Perrens, NutriNeuro (UMR INRAE 1286), University of Bordeaux, 33000 Bordeaux, France; 13 Pôle Psychiatrie, Centre Expert Dépression Résistante FondaMental, CHU La Conception, 13000 Marseille, France; 14 Département des Urgences et Post-Urgences Psychiatriques, Centre Expert Dépression Résistante FondaMental, CHRU Lapeyronie, 34000 Montpellier, France; 15 Service de Psychiatrie Adulte, Centre Expert Dépression Résistante FondaMental, CHRU de Lille, Hôpital Fontan 1, 59000 Lille, France; 16 Service de Psychiatrie de L’adulte, Centre Expert Dépression Résistante FondaMental, CHU de Brest, Hôpital de Bohars, 29820 Bohars, France; 17 UPEC, AP-HP Département Hospitalo-Universitaire d’Addictologie et de Psychiatrie (DMU IMPACT) des Hôpitaux Universitaires Henri Mondor, Centre Expert Dépression Resistante de la Fondation FondaMental, 94000 Créteil, France; 18 Amaris, 75009 Paris, France; sophie.marguet1@amaris.com (S.M.); nathalie.dennis@amaris.com (N.D.)

**Keywords:** treatment-resistant depression, real-world, utility, healthcare resource use

## Abstract

Background: Major depressive disorder (MDD) is among the most common psychiatric disorders. One-third of patients are usually unresponsive to several lines of treatment. This study aimed to describe the FondaMental French cohort of patients with treatment-resistant depression (TRD) and to estimate utility and healthcare resource use outcomes. Methods: Patients with TRD were evaluated prospectively over four years (baseline, 6, 12, 18, 24, 36 and 48 months) in a real-world clinical setting. Interim analyses focused on the first two consecutive years. Four MDD-related states (major depressive episode (MDE), response, remission, recovery) were defined based on the MADRS (Montgomery–Åsberg depression rating scale) and other clinical events. Health status was assessed with the EuroQol 5 Dimensions 5 Level (EQ-5D-5L) questionnaire. Utility values were estimated as preference measures that the patients assigned to their overall health status. Results: This study was based on 252 patients with TRD. The mean utility value by health state was 0.41, 0.63, 0.80, and 0.90, for MDE, response, remission, and recovery, respectively. At baseline, 59% of patients had an MADRS score of at least 28. Their baseline average utility value was lower compared to the other patients (0.43 versus 0.58, *p* < 0.001). This significant difference persisted at the following visits. The rate of patients in MDEs having at least one hospitalisation for depression or other reasons than depression was generally higher than that in the other health states. Conclusion: This study documented patterns in healthcare resource consumption, quality of life, and other characteristics in patients with TRD, both globally and by health state and depression severity.

## 1. Introduction

Major depressive disorder (MDD) is one of the most common psychiatric disorders, with a prevalence of 9.8% in France in 2017 [1]. It is estimated that one out of every three patients do not achieve an adequate response after several lines of treatment and will potentially progress to treatment resistance [2,3,4,5]. Due to the resilient nature of the disease and its burden, treatment-resistant depression (TRD) has become of particular interest in clinical research and public health. Multiple definitions of TRD exist, ranging from the non-response to a single adequate trial of an antidepressant to failing to respond to multiple antidepressants of different classes [6,7,8,9]. Formalised French recommendations by The French Association of Biological Psychiatry and Neuropsychopharmacology (AFPBN) and the Fondation FondaMental have defined TRD as the failure of at least two pharmacologically distinct antidepressant treatments used for an adequate duration (4–6 weeks) and at a sufficient dose (target dose reached) [10,11,12]. This is one of the most commonly used definitions in clinical practice, and several staging models exist to identify these characteristics [13].

It is estimated that TRD represents 15 to 30% of all depressive episodes [14]. It has been found that patients with MDD with greater depression severity and longer illness duration are more likely to be resistant to antidepressants, psychotherapy, and electroconvulsive therapy (ECT) [9]. Other risk factors include psychiatric comorbidities, such as anxiety and personality disorders, as well as substance abuse and undiagnosed or uncontrolled medical comorbidities [9,15]. TRD also incurs a substantial economic burden, with direct healthcare costs, resource use, and indirect costs estimated at twice as high compared to non-resistant MDD in the United States [16,17]. In France in 2015, the total direct and indirect costs of mental health problems was estimated at EUR 81 billion annually [18], and three out of four work-related mental disorders were due to depression [18].

Previous observational studies on TRD have been primarily retrospective using claims databases or electronic health records [7,16,19,20]. However, it is difficult to correctly identify patients with TRD using claims records given the multiple definitions of TRD and subjectivity in detecting an adequate duration and appropriate adherence to treatment. With fewer prospective cohorts of patients with TRD [21,22], there is a lack of data on this specific population. More real-world evidence is therefore needed to better understand TRD, to improve its diagnosis and management, and to inform cost-effectiveness studies.

This study was carried out using the FondaMental cohort, the first prospective cohort study on patients with TRD in France. The objectives of this study were to describe patients with TRD based on demographic and clinical variables, to evaluate healthcare resource use, to assess patients’ utility values, and to describe patients by the health states corresponding to the clinical course of the disease: major depressive episode (MDE), response, remission, and recovery.

## 2. Methods

### 2.1. Data Source

The FondaMental cohort is a French multicentre, prospective observational study of patients with TRD [23]. It is led by FondaMental, a public academic foundation created by the French Ministry of Research in 2007.

The FondaMental cohort includes patients from 13 French expert centres specialising in the management and treatment of MDD. Inclusions in the cohort began in September 2013, and 450 TRD patients are planned to be recruited [23]. An interim analysis was conducted on the cohort using a data cut-off date of 19 February 2018.

Patients were included in the cohort if they met the following criteria: experiencing an MDE according to the DSM-IV-TR (Diagnostic and Statistical Manual of Mental Disorders, 4th Edition, Text Revision) criteria [24]; failing at least two pharmacological treatments of two distinctly different classes (Stage II of the Thase and Rush staging model [25]); and experiencing moderate to severe depressive symptoms, as indicated by scores above 20 on the Montgomery–Åsberg Rating Scale (MADRS) [26,27]. Patients with bipolar disorders, psychotic disorders, obsessive compulsive disorders, eating disorders (with body mass index inferior to 15), somatoform disorders, and mood disorders related to substance abuse or misuse were systematically not included in the cohort.

The data collection process has been previously described [23]. In brief, data were collected through a web-based application, e-resistant depression©, which gathered all clinical monitoring, research and treatment data. Both self-administered (EQ-5D-5L) and observer-rating instruments (MADRS, Columbia-Suicide Severity Rating Scale (C-SSRS)) were used [23].

### 2.2. Study Design

This study provides an analysis of the prospective FondaMental cohort. The patients received an exhaustive clinical assessment at the date of inclusion in the cohort, which was assigned as the index date in this study. Follow-up assessments were performed every six months until the occurrence of one of the following: the last visit before death or drop-out; the last visit before data extraction; or the final visit at four years post-index date. Data were extracted on 19 February 2018.

### 2.3. Study Outcomes

Demographic and medical data were collected at the baseline evaluation. The severity of depressive symptoms was evaluated according to the MADRS score. Suicidal behaviour was measured using the C-SSRS [28].

The collection of treatment information relied primarily on patient-treating physician input, patient prescriptions, and patient records. In particular, antidepressant treatment use was analysed, as well as the use of ECT and repetitive transcranial magnetic stimulation (rTMS). Treatment proposals were made during baseline visits and each follow-up visit for patients who did not respond to the previously proposed treatment strategies.

Healthcare resource consumption was recorded at each visit by the occurrences between two consecutive visits. Hospitalisations were assessed both aggregately and by cause, i.e., if the hospitalisation was related to depression or not. Emergency room visits, ambulance rides, and outpatient visits by specialty were also reported.

As an assessment of health status, the EQ-5D-5L questionnaire [29], was completed by patients at each visit, assessing the severity of five different dimensions: mobility, self-care, usual activities, pain/discomfort, and anxiety/depression. Each dimension contains five severity levels: no problems, slight, moderate, severe, and extreme problems. The EQ-5D results for each dimension were converted into a single utility value using a mapping approach from the EQ-5D-5L to the EQ-5D-3L [30,31] and the French value set [32]. The single utility value aims to measure how health is valued by the general population of a country. Health state utility values represent strength-of-preference measures that individuals attribute to specific health-related outcomes. Utility measures are summarised by a score on a continuum scale which is generally between zero and one, with one corresponding to a perfect state of health and zero corresponding to death. In practice, utility values can be negative. These values therefore correspond to a health status “worse than death”.

The assessment protocol was approved by the institutional review board (French CNIL: DR-2015-673), in accordance with French law for non-interventional studies, and requires only an informational letter.

### 2.4. Statistical Analysis

Patient characteristics were described at baseline. Disease history and treatments at the inclusion in the cohort were also documented.

MDE, response, remission, and recovery were defined as health states corresponding to the clinical course of the disease. Health states were defined at each visit based on the MADRS score. MDE was defined as having an MADRS score of at least 28 at baseline, or of at least 22 at a follow-up visit. An MADRS total score of ≥28 was selected as the severity threshold to be consistent with the inclusion criteria of clinical trials conducted in a population of treatment-resistant patients [33,34]. Patients with an MDE at baseline were considered to be in response at a follow-up visit if they had at least a 50% reduction in their baseline MADRS score [35]. Remission was defined as an MADRS score ≤10 [36], and recovery was considered for patients after an uninterrupted duration in remission of six months. The MADRS score was assessed only every six months, therefore the uninterrupted duration in remission was assessed based on the MADRS scores at two consecutive visits and on the events that occurred between the two visits, as presented in Table 1 (first column).

Descriptive statistics for utility values and healthcare resource consumption were produced by health state. The EQ-5D-5L questionnaire was completed at each visit; the health states were therefore defined by the state at the actual visit for utility values. In the resource consumption analysis, however, the health states were defined for each interval between two consecutive visits. Healthcare resource consumption was also recorded at each visit, but it included all occurrences between two consecutive visits. For each interval between two visits, the health states were defined by the visit at the beginning of the interval and by the events occurring at or before the next visit, as presented in Table 1 (second column).

Subgroup analyses were conducted by disease severity based on the MADRS score at baseline (<28 and ≥28), by health state and by patients receiving ECT after inclusion in the cohort. Chi-squared tests and *t*-tests were performed to test for differences between the groups.

## 3. Results

The cohort included 252 patients at the time of this intermediate analysis. The number of patients completing six months, one year, 18 months and two years of follow-up was 162 (64%), 123 (49%), 81 (32%) and 78 (31%), respectively. At baseline, the average age was 53 years old, and about 63% of the patients were female. About 59% (143/244) of the population had a baseline MADRS score of at least 28. Over half of the patients had experienced suicidal thoughts prior to inclusion (52%), and 26% of patients had attempted suicide at least once in their lifetime (Table 2).

Among the patients reporting pharmacological treatment use at baseline or during follow-up, 83% (185/223) were treated with at least one antidepressant. During this time, venlafaxine (serotonin-norepinephrine reuptake inhibitor (SNRI)) was the most commonly reported antidepressant, used by 24% of the patients (44/185), followed by clomipramine (tricyclic antidepressant (TCA)), used by 22% (40/185) of the patients. The proportion of patients using ECT and rTMS within six months of baseline was 9% (19/206) and 4% (8/208), respectively. When including patients that used ECT and rTMS during the follow-up, the proportions rose to 23% (58/252) and 7% (18/252), respectively (Table 3).

The healthcare resource use and utility values by health state are presented in Table 4. Healthcare resource use was generally greater for patients in MDEs than in the other health states. Hospitalisations for depression were only present in MDEs because it served as a proxy to define relapse and recurrence. However, more patients with MDEs were also hospitalised for reasons other than depression (12%), while no patient in remission or recovery was hospitalised without depression during follow-up. Overall, there were few reports of ambulance rides and Emergency room (ER) visits. Utility values also differed depending on the state of health, with the lowest average utility value in MDE (0.41). These values increased with the improvement of MDD, and patients in response, remission, and recovery had an average utility value of 0.63, 0.80, and 0.90, respectively.

**Figure 1:**
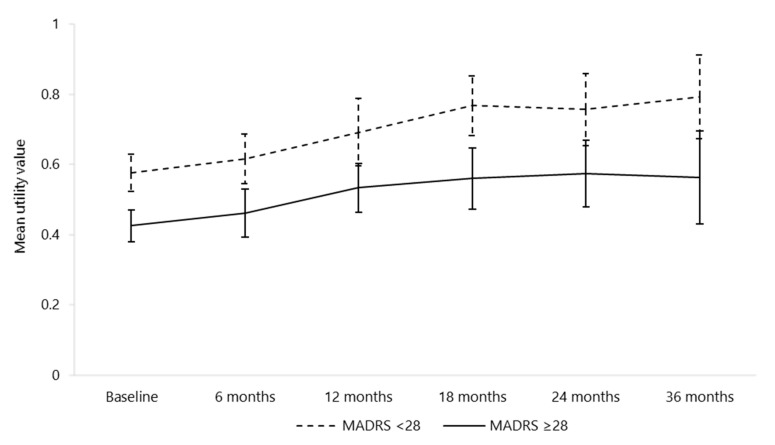
Mean utility values and confidence intervals by depression severity at each visit (*n* = 237 at baseline). MADRS: Montgomery–Åsberg Depression Rating Scale; *n:* number.

In more severe patients (baseline MADRS score of at least 28), the baseline average utility value was lower compared to the other patients (0.43 versus 0.58, *p* < 0.001). This significant difference subsisted at the following visits, as illustrated in Figure 1. The average utility values were 0.46 versus 0.62 (*p* < 0.01) at six months and 0.53 versus 0.69 (*p* < 0.01) at 12 months for the two groups, respectively. The difference between the two groups remained similar at the following visits. Between baseline and six months, 14 (23%) and 42 (42%) patients with a baseline MADRS score of less than 28 and of at least 28 were hospitalised for depression, respectively (*p* = 0.01). Other recorded healthcare resource use showed little difference among the depression severity groups.

**Figure 2:**
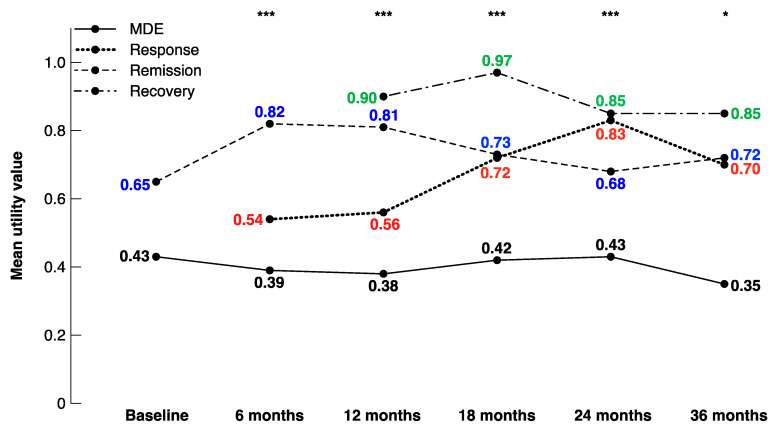
Mean utility values by health state at each visit. * *p* < 0.05, ** *p* < 0.01, *** *p* < 0.001.

The different health states were defined using the criteria explained in Table 1. As such, some patients were not designated a specific health state at certain visits. For example, patients with a baseline MADRS score between 10 and 28 were not categorised into a specific health state at baseline. In addition, although an MADRS score of at least 20 was required as an inclusion criterion, nine patients obtained an MADRS score of less than 10 at their baseline visit, and therefore were characterised as in remission (Table 5). In these depressed patients stratified on the basis of their health state, the utility values were consistently lowest among patients in MDEs and highest among patients in recovery, with averages ranging from 0.35–0.43 and 0.85–0.97, respectively. The utility values increased steadily among patients in response between 6 months (mean = 0.54) and 24 months (mean = 0.83). The utility values were significantly different among the health states during each follow-up year, as depicted in Figure 2.

Baseline patient characteristics by use of ECT post-inclusion in the cohort are presented in Table 6. Patients who were administered ECT were generally more severe at baseline compared to those receiving no ECT during follow-up. The subgroup analysis by ECT use during follow-up showed significant differences in hospitalisations for depression. Between baseline and six months, 34 (69%) patients that received ECT were hospitalised for depression compared to 24 (21%) patients that did not receive ECT during follow-up (*p* < 0.001), although other recorded healthcare use was more comparable among the groups. Utility levels were also similar between the two groups at baseline and during follow-up.

## 4. Discussion

Our study provides important findings on outcomes for patients with TRD. In addition to the lack of evidence on patients with TRD, there is even less information available on the course of the disease using both health states and the characteristics of patients in each health state. This work showed that TRD patients in MDEs had more hospitalisations unrelated to depression compared to those in response, remission, or recovery. In addition, results from the utility values indicated that patients’ health was lower in MDEs than in the other health states, and patients in recovery had a high average utility value (0.90). Moreover, the lower utility observed in more severe patients at the inclusion in the cohort subsisted during the follow-up. It was also found that patients with ECT during the follow-up had more severe diseases at baseline and had more hospitalisations for depression during follow-up.

The FondaMental cohort is the first prospective cohort on patients with TRD in France. In addition to collecting data on clinical measures, health status questionnaires were completed, and healthcare resource use was evaluated. Relevant health states were also identified, and utility values were obtained for these patients, both globally and by health state. Utility values, which measure health status in a single common metric, make it possible to compare patients’ health across populations and diseases. The use of health states also provided more detailed analyses on TRD, which used proxy variables to identify relapse, recurrence, and recovery. This approach made it possible to obtain more robust estimates by health state by taking into account events that characterise relapse or recurrence. Finally, patients in this cohort were followed up for four years, with 78 patients (31%) having at least two years of follow-up.

Healthcare resource use and utility values have also been analysed among patients with depression, but the literature is still sparse on patients with TRD. Amos et al. [16] identified patients with TRD from a U.S. commercial claims database and found that patients with TRD had twice the number of hospitalisation days when compared to non-TRD MDD patients. The Prospective Epidemiological Research on Functioning Outcomes Related to Major depressive disorder (PERFORM) study [37] followed European patients that were initiating antidepressant treatment their general practitioners. Among these patients, 2.5% with a depressive episode longer than 8 weeks were hospitalised within 12 weeks of the baseline visit. Our study had a higher percentage of patients hospitalised, with 32% of MDE patients hospitalised for depression between the baseline visit and the end of follow-up. The higher number of patients being hospitalised could be due to the difference in depression severity among the two cohorts; the patients in the PERFORM study were undergoing their first line of therapy rather than being treatment resistant. In addition, the differences in hospitalisations may also be related to patients being more closely monitored in an expert centre than by a primary care physician or psychiatrist.

The utility values found in this study were relatively similar to those reported in the Factors influencing depression endpoints research (FINDER) study [38]. The FINDER study was a European prospective observational study that evaluated quality of life outcomes in patients with depression and anxiety. Patients were followed from the beginning of an antidepressant treatment for up to six months, and results were available for the French subpopulation. Among 606 French patients treated with an antidepressant, they found an average utility value at baseline of 0.38, which increased to 0.68 and 0.75 after three months and six months of antidepressant treatment, respectively. Our study found that patients in MDEs had an average utility value of 0.41, and this value was higher for patients in improved health states. In two real-world studies, a utility value was estimated at 0.80 in 505 French patients with untreated chronic hepatitis C and at 0.67 in 1030 European patients with advanced non-small cell lung cancer [39,40]. Ara et al. [41] found that the average utility value for patients with mental illness, depression, or neurosis was 0.61. In the present study, patients with MDEs had a lower average utility value, which might be due to the fact that this study focused only on more severely ill patients with TRD. However, patients in recovery in the FondaMental cohort had a mean utility value of 0.90, indicating the improvement that can be made for patients with TRD, especially when followed closely at an expert centre.

Other studies have evaluated treatment strategies for patients with TRD. The Sequenced Treatment Alternatives to Relieve Depression (STAR*D)trial funded by the National Institute of Mental Health (NIMH) is the largest antidepressant study on MDD [4]. Randomisation was limited in that only acceptable treatment strategies were considered for each patient. In particular, patients with TRD were assigned to either tranylcypromine (non-selective and irreversible MAOI) or venlafaxine in combination with mirtazapine. Although venlafaxine was the most commonly used first antidepressant in the FondaMental cohort, MAOIs and mirtazapine were less well represented, even though they were among the chosen treatments for patients with TRD in the STAR*D trial.

In our study, there was a relatively low proportion of patients treated with at least one antidepressant, especially at baseline (65%). This may be due to the fact that patients with TRD could themselves have discontinued their medication if they perceived it as ineffective while expecting a new therapeutic strategy, as proposed at the initial visit.

This study also presented several limitations. Firstly, patients filled out their treatment use at each visit, which was not always recorded in chronological order or with start and end dates. This made it difficult to conduct robust analyses on treatment pattern use, especially given that the line of therapy was not documented. Secondly, there was a high number of missing values for several variables, which is a common constraint in observational studies. In particular, work productivity and certain baseline medical characteristics were not included in the analyses due to missing values. Additionally, there was a gradual decline in the number of participants during follow-up. The chosen period of data collection may not have enabled the patients to complete all visits after entering the study. However, this does not rule out the possibility that some patients could have been lost to follow-up in our real-world setting. In this context, the reasons for dropout were not specifically investigated. In addition, patients had a follow-up visit scheduled every six months, which presented challenges when defining a relapse, recurrence, and recovery. This is because a relapse and recurrence could happen at any point between two visits and because recovery is defined as an uninterrupted duration in remission. Proxy variables were therefore used to estimate these states. As such, the defined health states varied by endpoint. For measurements at each follow-up visit, the health state at that visit was used, whereas healthcare resource consumption took into account relapses and recurrences occurring between two visits to obtain the least biased results. Moreover, healthcare resource utilisation-related direct cost analyses were not performed and would merit receiving particular attention for further medico-economic research studies in our TRD population. Finally, the lack of a control group based on non-resistant depressed patients for comparison purposes is another limitation to our study.

## 5. Conclusions

This is the first prospective observational study to describe and evaluate patients with TRD managed within expert centres in France. This study documented patterns in healthcare resource consumption, quality of life, and other characteristics in patients with TRD, both globally and by health state and severity of depression. These findings add useful information to the limited available evidence on patients with TRD, and highlight the need for better prognoses and more well-adapted treatment plans. These results can also provide valuable material for health authorities to help populate cost-effectiveness models with real-world evidence. However, it should be noted that the care of these patients is specific to expert centres and may not be generalisable to all practitioners. As such, it would be beneficial to link this data to the French national health insurance information system (SNIIRAM), to obtain more complete information on the healthcare resource use and medical history of these patients. Further research is therefore required to better understand treatment patterns and other clinical characteristics of patients with TRD in order to significantly improve the quality of care and provide a more efficient management of these individuals [10].

## Figures and Tables

**Table 1:** Definitions of health states.

Health State	At Each Visit	For Each Interval between Two Consecutive Visits
Major depressive episode (MDE)	MADRS score ≥28 at baseline, or ≥22 at a follow-up visit	(1) MDE at one visit and no ≥50% reduction in baseline MADRS score at the following visit OR (2) Any health state at a visit and at least one of the following: - MADRS score ≥22 at following visit - Hospitalisation for depression between visits - Suicide attempt between visits - Antidepressant treatment change between visits - Use of ECT or rTMS after ending antidepressant treatment use ≥28 days between visits
Response	≥50% reduction in baseline MADRS score	Response at one visit and none of the following: - MADRS score ≥22 at following visit - Hospitalisation for depression between visits - Suicide attempt between visits - Antidepressant treatment change between visits - Use of ECT or rTMS after ending antidepressant treatment use ≥28 days between visits
Remission	MADRS score ≤10	Remission at one visit and none of the following: - MADRS score ≥22 at following visit - Hospitalisation for depression between visits - Suicide attempt between visits - Antidepressant treatment change between visits - Use of ECT or rTMS after ending antidepressant treatment use of ≥28 days between visits
Recovery	MADRS score ≤10 for two consecutive visits, and none of the following between the two visits: - Hospitalisation for depression - Suicide attempt - Antidepressant treatment change - Use of ECT or rTMS after ending antidepressant treatment use ≥28 days	Recovery at one visit and none of the following: - MADRS score ≥22 at following visit - Hospitalisation for depression between visits - Suicide attempt between visits - Antidepressant treatment change between visits - Use of ECT or rTMS after ending antidepressant treatment use ≥28 days between visits

ECT: electroconvulsive therapy; MADRS: Montgomery–Åsberg Depression Rating Scale; rTMS: repetitive transcranial magnetic stimulation.

**Table 2:** Baseline demographic and clinical characteristics of patients in the FondaMental cohort.

Demographic Characteristics		*n* Missing
Number of patients	252	
Age in years, mean ± SD (median)	53.1 ± 13.0 (53.5)	0
Sex, *n* (%)		
Female	159 (63.1%)	0
Male	93 (36.9%)	
Education, *n* (%)		0
Some secondary education	95 (37.7%)	
Associate degree	50 (19.8%)	
At least some college	107 (42.5%)	
Employment status, *n* (%)		23
Employed	80 (34.9%)	
Unemployed	44 (19.2%)	
Retired	61 (26.6%)	
Student	4 (1.7%)	
Pension	15 (6.6%)	
Stay-at-home	9 (3.9%)	
Other	16 (7.0%)	
Marital status, *n* (%)		23
Single	46 (20.1%)	
Married/Civil Union	142 (62.0%)	
Separated	14 (6.1%)	
Divorced	24 (10.5%)	
Widowed	3 (1.3%)	
Household size, *n* (%)		38
1	66 (30.8%)	
2	84 (39.3%)	
3	28 (13.1%)	
>3	36 (16.8%)	
**Clinical Characteristics**		
MADRS, mean ± SD (median)	29.0 ± 6.9 (29.0)	8
Disease severity according to MADRS, *n* (%)		8
MADRS <28	101 (41.4%)	
MADRS ≥28	143 (58.6%)	
Lifetime suicide severity, *n* (%)		0
Wish to be dead	168 (66.7%)	
Non-specific active suicidal thoughts	130 (51.6%)	
Thoughts about the way in which to commit suicide	103 (40.9%)	
Suicidal thoughts with intention to follow-through	59 (23.4%)	
Construction of detailed scenario on how to commit suicide	44 (17.5%)	
Attempted suicide	65 (25.8%)	
Number of suicide attempts, mean ± SD (median)		
Entire cohort	0.5 ± 1.2 (0.0)	
Patients that attempted suicide at least once	1.9 ± 1.7 (1.0)	

MADRS: Montgomery–Åsberg Depression Rating Scale; N: number; SD: Standard Deviation.

**Table 3:** Antidepressant and non-pharmacological therapies used at baseline and during follow-up in the cohort.

Treatment Class/Treatment	*n* (%)	*n* Missing
**Antidepressants**		
Treated at baseline or during follow-up	185 (83.0%)	29 *
Treated at baseline only (among treated patients)	97 (64.7%)	35
First treatment recorded in the cohort		
Serotonin–norepinephrine reuptake inhibitors (SNRIs)	54 (29.2%)	
Venlafaxine	44 (23.8%)	
Duloxetine	10 (5.4%)	
Tricyclic antidepressants (TCA)	49 (26.5%)	
Clomipramine	40 (21.6%)	
Amitriptyline	6 (3.2%)	
Maprotiline	2 (1.1%)	
Imipramine	1 (0.5%)	
Selective serotonin reuptake inhibitors (SSRIs)	45 (24.3%)	
Fluoxetine	20 (10.8%)	
Sertraline	12 (6.5%)	
Paroxetine	10 (5.4%)	
Escitalopram	3 (1.6%)	
Alpha-2 antagonists	24 (13.0%)	
Mirtazapine	18 (9.7%)	
Mianserine	6 (3.2%)	
Reversible inhibitor of monoamine oxidase A (RIMA)	6 (3.2%)	
Moclobemide	6 (3.2%)	
Non-selective, irreversible monoamine oxidase inhibitor (MAOI)	3 (1.6%)	
Iproniazid	3 (1.6%)	
Other	4 (2.2%)	
Agomelatine	4 (2.2%)	
**Electroconvulsive therapy (ECT)/repetitive transcranial magnetic stimulation (rTMS)**		
Treated at baseline		
Overall	19 (9.2%)/8 (3.8%)	46/44
Patients with no antidepressant at baseline (*n* = 91)	4 (4.7%)/2 (2.4%)	6/7
Treated at baseline or during follow-up		
Overall	58 (23.0%)/18 (7.1%)	0
Patients with no antidepressant at baseline or follow-up (*n* = 38)	10 (26.3%)/3 (7.9%)	0

* More missing data for patients treated as baseline due to missing start dates of pharmacological treatments. *n* = number.

**Table 4:** Healthcare resource use and utility values by health state.

Healthcare Resource Use	MDE (*n* = 278)	Response (*n* = 22)	Remission (*n* = 38)	Recovery (*n* = 19)
Hospitalisations (all)				
Number of hospitalisations, mean (SD)	1.1 (3.2)	0.1 (0.2)	0 (0.0)	0 (0.0)
Patients with at least one hospitalisation, *n* (%)	106 (38.1)	1 (4.6)	0 (0.0)	0 (0.0)
Hospitalisations for depression				
Number of hospitalisations, mean (SD)	0.9 (3.2)	0 (0.0)	0 (0.0)	0 (0.0)
Patients with at least one hospitalisation, *n* (%)	89 (32.0)	0 (0.0)	0 (0.0)	0 (0.0)
Hospitalisations outside depression				
Number of hospitalisations, mean (SD)	0.2 (0.6)	0.1 (0.2)	0 (0.0)	0 (0.0)
Patients with at least one hospitalisation, *n* (%)	32 (11.5)	1 (4.6)	0 (0.0)	0 (0.0)
At least one ER visit, *n* (%)	20 (7.2)	1 (4.6)	2 (5.3)	2 (10.5)
At least one ambulance ride, *n* (%)	9 (3.2)	0 (0.0)	0 (0.0)	0 (0.0)
Utility values	MDE (*n* = 341)	Response (*n* = 49)	Remission (*n* = 91)	Recovery (*n* = 40)
Mean (SD)	0.41 (0.29)	0.63 (0.24)	0.80 (0.21)	0.90 (0.15)

MDE: major depressive episode; *n* = number; SD: standard deviation.

**Table 5:** Number of depressed patients by health state at each visit.

	MDE	Response	Remission	Recovery
Baseline	143	0	9	0
6 months	86	7	32	0
12 months	49	14	28	9
18 months	30	11	18	10
24 months	25	7	17	18
36 months	9	7	7	10
48 months	2	3	4	2

MDE: major depressive episode.

**Table 6:** Patient characteristics and results by use of ECT post-inclusion in the cohort.

	No ECT (*n* = 203)	ECT (*n* = 49)	*p*-Value
Patient characteristics at baseline			
Age in years, mean ± SD (median)	52.7 ± 13.2 (53.1)	54.9 ± 12.4 (55.7)	0.290
Female, *n* (%)	132 (65.0%)	27 (55.1%)	0.196
Disease duration (years), mean ± SD (median)	18.7 ± 12.5 (17.6)	16.1 ± 12.0 (17.2)	0.304
Chronic episode (>2 years), *n* (%)	66 (62.3%)	14 (46.7%)	0.125
Number of MDE resistant to ≥2 treatments, mean ± SD (median)	1.3 ± 1.0 (1.0)	1.2 ± 0.7 (1.0)	0.655
Number of MDE with psychotic characteristics, mean ± SD (median)	0.1 ± 0.5 (0.0)	0.2 ± 0.4 (0.0)	0.585
MADRS, mean ± SD (median)	28.4 ± 6.9 (28)	31.4 ± 6.8 (32)	0.007
Baseline MADRS ≥28, *n* (%)	107 (52.7%)	36 (73.5%)	0.009
Results			
Number of hospitalisations for depression, mean ± SD (median)			
Between baseline and 6 months	0.4 ± 1.4 (0)	2.8 ± 6.5 (1)	<0.001
Between 6 months and 1 year	0.4 ± 1.9 (0)	1.2 ± 1.8 (1)	0.030
At least one hospitalisation for depression, *n* (%)			
Between baseline and 6 months	24 (21.2%)	34 (69.4%)	<0.001
Between 6 months and 1 year	11 (13.4%)	24 (58.5%)	<0.001
Number of hospitalisations outside depression, mean ± SD (median)			
Between baseline and 6 months	0.1 ± 0.3 (0)	0.2 ± 0.5 (0)	0.207
Between 6 months and 1 year	0.1 ± 0.4 (0)	0.2 ± 0.6 (0)	0.698
At least one hospitalisation outside depression, *n* (%)			
Between baseline and 6 months	16 (14.2%)	5 (10.2%)	0.491
Between 6 months and 1 year	8 (9.8%)	4 (9.8%)	0.999
At least one ER visit during first year of follow-up, *n* (%)	8 (9.8%)	9 (22.0%)	0.065
At least one ambulance ride during first year of follow-up, *n* (%)	4 (4.9%)	4 (9.8%)	0.301
Utility values, mean ± SD (median)			
Baseline	0.49 ± 0.26 (0.51)	0.48 ± 0.33 (0.53)	0.799
6 months	0.51 ± 0.33 (0.53)	0.55 ± 0.31 (0.58)	0.416
1 year	0.59 ± 0.32 (0.67)	0.60 ± 0.30 (0.56)	0.876

ECT: electroconvulsive therapy; ER: emergency room; MDE: major depressive episode; n = number; SD: standard deviation.

## Abbreviations

Major depressive disorder (MDD); treatment-resistant depression (TRD); major depressive episode (MDE); Montgomery–Asberg Depression Rating Scale (MADRS); The French Association of Biological Psychiatry and Neuropsychopharmacology (AFPBN); electroconvulsive therapy (ECT); Columbia-Suicide Severity Rating Scale (C-SSRS); repetitive transcranial magnetic stimulation (rTMS); serotonin-norepinephrine reuptake inhibitor (SNRI); tricyclic antidepressant (TCA); selective serotonin reuptake inhibitors (SSRIs); reversible inhibitor of monoamine oxidase A (RIMA); monoamine oxidase inhibitor (MAOI).
